# Severe symptomatic hyponatremia due to cerebral salt wasting syndrome in a patient with traumatic head injury and Dandy-Walker malformation of the brain 

**DOI:** 10.5414/CNCS110146

**Published:** 2021-02-19

**Authors:** Orfeas Liangos, Nicolaos E. Madias

**Affiliations:** 1 KfH Nierenzentrum Lichtenfels,; 2 University of Würzburg Faculty of Medicine, Würzburg, Germany, and; 3 St. Elizabeth’s Medical Center, Department of Medicine, Tufts University School of Medicine, and; 4 Tufts University School of Medicine, Boston, MA, USA

**Keywords:** hyponatremia, salt wasting, cerebral, Dandy-Walker malformation

## Abstract

Cerebral salt wasting (CSW) is an uncommon cause of hyponatremia characterized by extracellular volume depletion, high urine sodium concentration and osmolality, and low serum uric acid concentration in association with central nervous system (CNS) disease. Distinguishing CSW from the syndrome of inappropriate secretion of antidiuretic hormone (SIADH), a much more common form of hyponatremia in this setting, can be challenging because both present with identical laboratory features. However, treatment of CSW and SIADH differs, making a correct diagnosis important. Here we present a case of CSW in a 75-year-old man in whom severe hyponatremia and volume depletion were discovered in the setting of traumatic head injury and Dandy-Walker malformation of the brain, a rare congenital brain malformation. Treatment with intravenous normal saline and later oral salt supplementation and fludrocortisone was successful.

## Introduction 

Hyponatremia is a common and potentially serious condition in community-dwelling and hospitalized patients [1, 2, 3]. Correct differential diagnosis is essential to sound treatment but can be very challenging [4]. Appropriate treatment, tailored to the causative pathomechanism, restores normonatremia while preventing potentially life-threatening complications caused by the disturbance itself or its management [4]. The following case report illustrates how timely and systematic differential diagnosis resulted in the identification of cerebral salt wasting (CSW), an uncommon cause of hyponatremia, thereby ensuring correct management. The unique features of the presented case are then discussed in the context of existing literature. 

## Case presentation 

A 75-year-old man presented to the emergency department of Klinikum Coburg after falling on his head while using a walker in the nursing home. He had been residing at that facility for ~ 3 weeks following a hospitalization in the surgical service of Klinikum Coburg for a traumatic right-sided parietal skull impression fracture. The head injury had resulted from a fall on the occipital area of the head with loss of consciousness, while the patient was still living independently in his own home. Although hyponatremia had been noted during that hospitalization (see below), the nephrology service was not consulted and no specific treatment was prescribed. 

At the time of the current presentation, the patient reported dizziness but denied loss of consciousness; he also had no shortness of breath, chest pain, diarrhea, vomiting, or fever. The initial blood pressure in the emergency room was 98/50 mmHg with a heart rate of 82/min. The Glasgow Coma Scale was 15, and there were no focal neurological deficits. A fresh, bleeding laceration of the skin at the right temple was present. The mucous membranes were dry, and no jugular venous distention was noted; lungs were clear to auscultation bilaterally, skin turgor was reduced, and there was no peripheral edema. The remainder of the physical examination was normal. The skin laceration required suturing for hemostasis and wound closure. On questioning, the patient described that he had fallen several times bumping his head, even sustaining a bleeding head wound, in the days leading up to his first hospitalization, and that he had increased his fluid intake to counteract orthostatic dizziness. Past medical history included arterial hypertension and type 2 diabetes mellitus for which the patient was taking candesartan and metformin. He had no history of chronic renal disease and denied use of diuretics. 

## Investigations 

The admission laboratory examination showed a serum sodium concentration of 118 meq/L, potassium 3.6 meq/L, creatinine 0.9 mg/dL, urea nitrogen 7 mg/dL, glucose 101 mg/dL, and measured osmolality 226 mosm/kg. The thyroid-stimulating hormone was normal. Urine sodium and osmolality 10 hours following admission were 83 meq/L and 518 mosm/kg, respectively. The serum uric acid concentration 1 day following admission was 2.6 mg/dL. At the previous hospitalization ~ 3 weeks earlier, hyponatremia had already been present with an initial serum sodium of 119 meq/L. 

**Figure 1:**
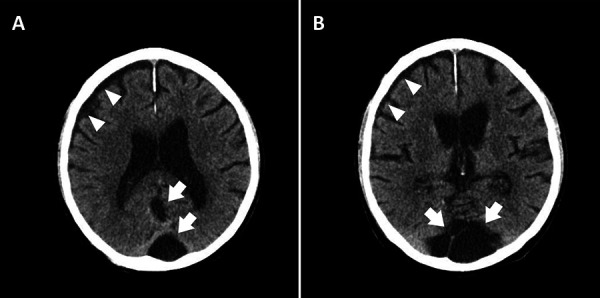
Computertomographic axial cross sections of the brain without contrast. Frontotemporal hygroma (arrowheads); Dandy-Walker cyst (arrows).

A CT scan of the head on admission, without administration of contrast agents, showed no brain edema or midline shift, and no signs of fresh intracranial hemorrhage. Compared to a previous examination ~ 4 weeks earlier, a newly developed right frontal hygroma was noted (Figure 1, arrowheads). The known, right-sided parietal impression fracture of the skull was unchanged. In addition, a previously diagnosed Dandy-Walker cyst in the posterior fossa was also unchanged (Figure 1, arrows). 

**Figure 2:**
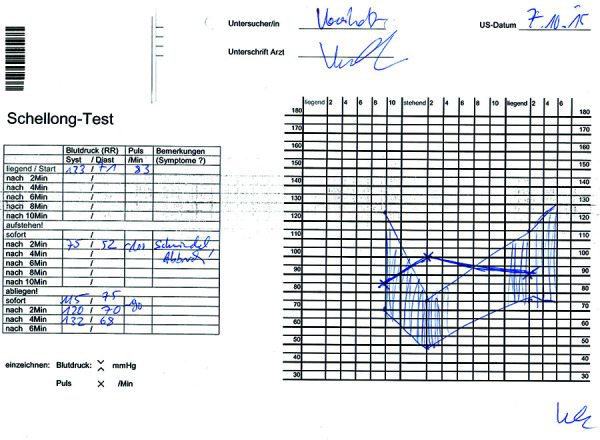
Diagram of a Schellong test obtained during the course of inpatient treatment. Pulse rate is indicated by an “x”; systolic and diastolic blood pressure by a “.” in the diagram. Note the profound orthostatic decrement in blood pressure associated with a concomitant increase in heart rate.

On the 6^th^ day of admission, with the serum sodium concentration at 129 meq/L, an orthostatic blood pressure test was performed. While supine, blood pressure and pulse were 123/71 mmHg and 83/min, respectively; following 2 minutes of orthostasis, the values changed to 75/52 mmHg and 100/min, respectively. The test was aborted due to dizziness and lightheadedness. Immediately after returning to the supine position, the blood pressure and pulse recovered to 115/75 mmHg and 90/min, respectively (Figure 2). 

## Differential diagnosis 

Hyponatremia has a broad differential diagnosis that includes iso-, hypo- and hyperosmolar forms. The measurement of serum osmolality, which occurred at admission to the hospital, although lower compared to the calculated osmolality (2 × serum Na + glucose (mg/dL)/18 + blood urea nitrogen (mg/dL)/2,8) and in this case 244 mosm/kg, shows no significant osmolar gap. These findings identify the case as a hypoosmolar hyponatremia and rule out the presence of an osmolar gap. 

The next step is the evaluation of the volume status of the patient. The low blood pressure, orthostatic hypotension (Figure 2), decreased skin turgor, and absence of peripheral edema identify the volume status as hypovolemic. Additional imaging examinations, such as a chest X-ray with clear lung fields, a normally sized heart shadow, and a transthoracic echocardiogram with normal-sized heart cavities and normal left-ventricular ejection fraction rule out congestive heart failure and hypervolemia. 

Further, the elevated urine osmolality is consistent with antidiuretic hormone (ADH)stimulation and water reabsorption in the collecting tubules, resulting in a concentrated urine. Hypovolemia can result from nonrenal or renal sodium losses. If the urinary sodium is higher than 40 meq/L in a patient with hypovolemia, it points to renal salt wasting. In a patient with cerebral pathology, hypovolemia, and renal salt wasting in the absence of diuretics, chronic renal disease (particularly the interstitial type), and aldosterone deficiency (see below), cerebral salt wasting emerges as the most plausible cause of the prevailing hyponatremia. The low serum uric acid concentration in our patient is also consistent with a cerebral salt wasting condition. 

## Treatment 

The patient was transferred to the nephrology service of our hospital and prescribed oral fluid restriction of 1 L per day. Under administration of 2 L of 0.9% normal saline daily for the first 4 days, the serum sodium increased to 122 meq/L on day 2, 126 meq/L on day 3, and 130 meq/L on day 5. In parallel, the urine sodium concentration increased further to 165 – 170 meq/L and the urine osmolality ranged between 433 and 456 mosm/kg. Starting on day 6, 1,000 mg sodium-chloride tablet along with 1 scoop of protein powder were prescribed thrice daily and fludrocortisone 0.1 mg once daily. The patient was discharged on this medication regimen along with bilateral knee-high compression stockings. Serum and urine Na at discharge were 132 and 24 meq/L, respectively. Medication adherence after discharge was ensured by the nursing-home staff, where the patient resided permanently. 

## Outcome and follow-up 

**Figure 3:**
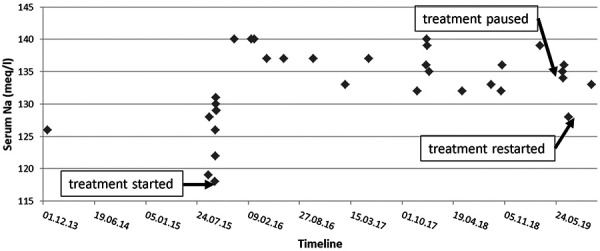
Diagram of the serum sodium concentration over time. Initiation, discontinuation, and re-start of treatment for cerebral salt wasting are indicated by arrows. Note the chronicity of hyponatremia before treatment was started, maintenance of normonatremia under treatment with fludrocortisone and sodium chloride supplementation, and re-occurrence of hyponatremia when treatment was paused.

Additional laboratory and imaging tests were performed as an outpatient. A CT scan of the chest showed no lung parenchymal masses or nodules. Serum cortisol was 62 µg/L, serum aldosterone 16 ng/L, and plasma renin concentration 2.2 ng/L with an aldosterone-to-renin ratio of 7.3 (all normal). While under treatment with fludrocortisone, these results rule out adrenal insufficiency and hypoaldosteronism as potential causes of orthostatic hypotension, salt wasting, and hyponatremia. The patient was seen in the outpatient nephrology clinic in follow-up 2, 4, and 8 months after discharge from the hospital. He reported feeling well and denied further falls; his blood pressure was measured in our clinic and found to be in the normal or mildly elevated range without orthostatic symptoms. His serum sodium on the above treatment continued to range between 132 and 140 meq/L over 4 years of follow-up (Figure 3), while the patient’s clinical condition remained stable. Fludrocortisone and salt supplementation were then discontinued, but at 1 month of follow-up, serum Na fell to 128 meq/L with a urine sodium of 76 meq/L, and the patient reported orthostatic dizziness and tendency to fall. Fludrocortisone and oral salt were restarted with resolution of symptoms and increase in serum Na to 133 meq/L. 

## Discussion 

By far the majority of hyponatremia in the setting of central nervous system (CNS) disease is attributed to syndrome of inappropriate secretion of antidiuretic hormone (SIADHI with only a small fraction being assigned to CSW [5]. Most cases of CSW involve patients with subarachnoid hemorrhage (SAH), but it has also been observed in association with other CNS pathologies, including infections, intrinsic and metastatic tumors, neurosurgical procedures, and trauma [5, 6, 7]. Although SIADH and CSW share common laboratory findings, such as high urine sodium and low serum uric acid concentrations, their key distinguishing feature is the volume status. However, serum uric acid might help in distinguishing CSW from other forms of hyponatremia due to volume depletion, in which serum uric acid is expected to be elevated as a reflection of a prerenal state. SIADH is a euvolemic state, hyponatremia being generated by electrolyte-free water retention due to the inappropriate release of ADH. By contrast, CSW is characterized by hypovolemia caused by renal salt and water losses; in turn, hypovolemia stimulates ADH release that generates the hyponatremia. Natriuretic peptides, such as brain-type natriuretic peptide and endogenous digitalis-like factors, have been implicated in the pathogenesis of natriuresis and volume depletion in CSW [5, 6, 8]. The difficulty in distinguishing SIADH from CSW lies primarily in correctly assessing volume status, due to the lack of reliable measurement methods [8, 9]. The clinical evaluation of the patient remains central in the assessment of volume status but can be a frequent source of error. Some authors therefore have doubted the existence of CSW altogether and attribute the hyponatremia to SIADH, whereas the increased natriuresis and negative sodium balance are thought to result from prior copious infusions of isotonic saline causing so-called “overshoot natriuresis” [8, 9]. Furthermore, volume depletion could reflect pressure natriuresis due to the commonly observed adrenergic surge in patients with CNS injury that results in increased arterial blood pressure and contraction of venous capacitance vessels [8, 9]. Nevertheless, the existence of CSW has been supported by several studies, in which volume status was measured in adult neurosurgical patients with hyponatremia using radioisotope dilution; in such studies, volume depletion, rather than euvolemia, was detected and therefore hyponatremia was attributed to CSW rather than SIADH [10]. 

SIADH and CSW require distinct therapeutic approaches. When mildly or moderately symptomatic, the former can be treated with fluid restriction, loop diuretics and increased salt intake, increased solute intake (salt, protein), urea, or a vasopressin receptor antagonist. Administration of isotonic sodium chloride will routinely cause aggravation of the hyponatremia. When severely symptomatic, SIADH requires administration of hypertonic saline. By contrast, CSW requires administration of isotonic sodium chloride for volume repletion, and then supplemental oral salt and, occasionally, mineralocorticoids for as long as the salt wasting persists. The clear establishment of the presence of hypovolemia in our patient and the correction of his hyponatremia with volume repletion leave no doubt that his hyponatremia was not caused by SIADH. Measurement of urine sodium can be helpful as well, distinguishing CSW from non-renal causes of volume depletion, with a urine sodium concentration expected to be elevated in CSW and low in the latter. However, we note that in our case, urine sodium was collected 10 hours after admission in which time between 500 and 1,000 mL normal saline had already been administered; this could lead to elevated urine sodium even in the setting of hypovolemia and increased tubular sodium avidity. Later in the clinical course of the presented case, the persistence of a relatively high plasma renin concentration of 2.2 ng/L despite concomitant treatment with fludrocortisone and salt tablets provides additional evidence for salt wasting that is preventing the development of volume expansion, further supporting the diagnosis of CSW. 

Regarding the associated cause, we cannot attribute the CSW in our patient to head trauma from falls occurring prior to and subsequent to his first hospitalization at our institution, because we were able to document that moderately severe hyponatremia was evident years before these injuries happened (Figure 3). In particular, the patient visited the emergency room of our hospital in 2013 after a fall with head laceration, when a single serum sodium of 126 meq/L was measured. At that time, no diagnostic or therapeutic efforts were made regarding the hyponatremia as the patient was not seen by internal medicine or nephrology. More remote serum sodium values were not available. In addition, when treatment with oral salt and fludrocortisone (which had successfully maintained stable serum sodium values for ~ 4 years) was discontinued in 2019, hyponatremia and renal salt wasting reappeared in the absence of preceding head trauma (Figure 3). Rather than being causative, the patient’s falls and associated head injuries might have been consequences of the gait instability characteristic of hyponatremia [11, 12]. This notion is supported by emergency room presentations of the patient for falls in the years 2010 and 2013, preceding the hospitalization in 2015 that is presented in this article. Furthermore, the patient by his own account has reported that he had been experiencing gait instability and orthostatic dizziness for several years prior to the current event which had improved in a substantial and sustained fashion since therapy with oral salt and fludrocortisone was instituted. However, we cannot exclude the possibility that repeated head trauma intensified the patient’s salt wasting thereby contributing to his hyponatremia at the time of his hospitalizations. 

We contend that the patient’s Dandy-Walker malformation of the brain is the underlying cause of his CSW. First described by Dandy and Blackfan [13] and subsequently by Taggart and Walker [14], the Dandy-Walker malformation involves a cystic malformation in the posterior fossa and hypoplasia of the cerebellar vermis, and is frequently associated with subsequent development of internal hydrocephalus [15]. This rare disorder can occur in the company of other congenital anomalies of the heart, large vessels, face, and jaw [15, 16]. Although associated with high early mortality ranging between 12.5 and 50%, neurosurgical treatment is not always indicated in milder forms, and an intelligence quotient of ≥ 80% is observed in 29 – 67% of patients. Older individuals with the condition can present with signs resembling a posterior fossa tumor, or the diagnosis is made incidentally during imaging studies [16]. To our knowledge, cases of Dandy-Walker malformation in association with hyponatremia have not previously been reported. However, case reports of other congenital CNS anomalies, such as agenesis of the corpus callosum [17, 18] and Rathke’s cleft cyst [19, 20] exist that have been associated with chronic hyponatremia attributed to SIADH but not CSW. 

Given the congenital nature of the Dandy-Walker malformation, it is likely that our patient has had hyponatremia much earlier in life, the timing of appearance determined by the magnitude of his renal salt wasting in relationship with his salt intake. Progression of the structural consequences of the malformation in the course of life might trigger functional consequences aggravating the renal salt wasting. If our contention is correct, it remains to be defined in which ways the Dandy-Walker malformation of the brain can induce sustained natriuresis and volume depletion thereby generating chronic hyponatremia. 

## Teaching points

- We propose that the present report represents the first description of chronic cerebral salt wasting (CSW) requiring long-term treatment. It is attributed to a Dandy-Walker malformation of the brain, a previously unrecognized cause of hyponatremia.
- CSW should be considered in patients with central nervous system pathology who develop hyponatremia associated with signs and symptoms of hypovolemia and elevated urinary sodium excretion.
- Before making the diagnosis, other causes of hyponatremia with elevated urine sodium should be carefully excluded.
- Special attention should be given to the assessment of volume status, frequently the only feature to distinguish CSW from the syndrome of inappropriate antidiuretic hormone secretion (SIADH), for example by using multiple means of assessment (clinical and laboratory assessment, and ultrasound, echocardiographic, or chest X-ray imaging).
- Treatment may include intravenous or oral salt loading, mineralocorticoids and perhaps a mild oral fluid restriction.

## Funding 

The authors received no financial support for this work. 

## Conflict of interest 

The authors report no conflict of interest, financial or otherwise, pertaining to this manuscript.
